# Gastric venous congestion after pancreatic surgery: A systematic review, metanalysis and suggested protocol for assessment and management

**DOI:** 10.1007/s00423-026-04049-8

**Published:** 2026-04-25

**Authors:** Krishna Kotecha, Hiro Masuda, Krupa Kotecha, Sanjay Pandanaboyana, Koroush S. Haghighi, Charbel Sandroussi, Matthew Katz, Anubhav Mittal, Jaswinder Samra

**Affiliations:** 1 Royal North Shore Hospital, Reserve Road, Sydney, 2065 NSW Australia; 2 The University of Sydney, Sydney, NSW Australia; 3 Newcastle University, Newcastle Upon Tyne, UK; 4 University of New South Wales, Kensington, NSW Australia; 5 Prince of Wales Hospital, Randwick, NSW Australia; 6 Royal Prince Alfred Hospital, Camperdown, NSW Australia; 7 The University of Texas MD Anderson Cancer Centre, Houston, TX USA; 8 University of Notre Dame, Sydney, Australia

**Keywords:** Pancreatic surgery, Gastric venous congestion

## Abstract

**Background:**

Gastric venous congestion (GVC) is an under-recognised complication of pancreatic surgery, particularly in the context of venous resection and splenectomy. This systematic review aimed to evaluate the incidence, clinical impact, and current strategies for diagnosis, prevention, and management of GVC.

**Methods:**

A PRISMA-compliant systematic review was conducted across MEDLINE, Embase, Cochrane Library, and additional sources (2005–2024). Studies reporting on GVC in pancreatic surgery were included. Data on surgical characteristics, incidence of intraoperative and postoperative GVC, and clinical outcomes were extracted. A single-arm meta-analysis of proportions was performed for outcomes reported in ≥5 studies using a random-effects model.

**Results:**

Sixteen studies including 1,133 patients were analysed. Intraoperative GVC was reported in 5.0–27.9% of cases, while postoperative GVC occurred in up to 24.1%. Meta-analysis demonstrated a pooled intraoperative GVC incidence of 16.3% (95% CI 9.8–25.9; I²=81.0%) and postoperative GVC incidence of 4.7% (95% CI 1.3–15.4; I²=83.3%). GVC was associated with increased morbidity, including delayed gastric emptying (pooled 17.3%, 95% CI 13.2–22.4), major complications (13.5%, 95% CI 8.1–21.6), and postpancreatectomy haemorrhage (4.5%, 95% CI 2.7–7.5). Ninety-day mortality was 4.3% (95% CI 2.9–6.5). Preservation or reconstruction of gastric venous drainage pathways was consistently associated with reduced postoperative GVC in reported series, although evidence was limited to non-comparative studies.

**Conclusion:**

GVC is a relatively common intraoperative finding and is associated with clinically significant postoperative morbidity. Pooled incidence estimates highlight substantial heterogeneity, reflecting variation in definitions and diagnostic approaches. Current evidence is predominantly retrospective and non-comparative; we therefore propose a potential standardised diagnosis and management pathway, of which prospective evaluation is required.

**Supplementary Information:**

The online version contains supplementary material available at 10.1007/s00423-026-04049-8.

## Introduction

 The widespread adoption of neoadjuvant therapy for pancreatic ductal adenocarcinoma (PDAC) has led to more advanced disease presentations at the time of surgery, often necessitating extended resections involving critical vascular structures. Porto-mesenteric vein resection, once reserved for select cases, is now performed in up to 40% of patients undergoing pancreatectomy [1]. Although these technical advancements have improved outcomes, gastric venous congestion (GVC), which occurs due to disruption of the critical venous drainage of the stomach, is now an understudied contributor to postoperative morbidity, including delayed gastric emptying (DGE), gastric ischemia and venous infarction.

This venous drainage system is primarily composed of three major pathways: the left gastric vein (LGV; also known as the coronary vein), the right gastric vein, and the gastroepiploic veins. The LGV drains the upper body and fundus of the stomach into the portal vein (PV). Additional venous outflow is provided by the short gastric veins and left gastroepiploic vein, which drain into the splenic vein (SpV), while the right gastroepiploic vein drains into the superior mesenteric vein. During pancreatoduodenectomy (PD) or total pancreatectomy, particularly when combined with splenectomy or portomesenteric venous resection, these critical venous pathways may be disturbed [2]. Collateral pathways are often insufficient to drain the remnant stomach, resulting in elevated venous pressure and stasis in the gastric veins, which can lead to edematous congestion and impaired oxygen delivery [1, 3]. This can impair the contractile function of the stomach and disrupt the surgical anastomotic sites. Elevated intragastric pressure due to venous stasis can also interfere with coordinated peristalsis in the stomach and duodenum. These pathophysiological changes collectively contribute to delayed transit of gastric contents into the small intestine, causing DGE [2, 3]. The risk of subsequent ischemia is particularly high when key collateral veins such as the gastroepiploic veins or short gastric veins are also sacrificed or fail to provide adequate compensatory drainage. Furthermore, anatomical variations in venous termination—such as whether the LGV drains into the portal vein or splenic vein—can influence susceptibility to GVC during surgery [1]. Finally, disruption of the splenic vein drainage is known to be associated with left sided portal hypertension (sinistral hypertension) with associated splenic varices [4], and their associated complications, including bleeding and splenomegaly.

To mitigate GVC and its complications, various strategies have been proposed. These include preserving key venous structures whenever possible, reconstructing venous outflow pathways intraoperatively, or performing subtotal/total gastrectomy during the index procedure. However, there remains a lack of comprehensive evaluation of these approaches. This systematic review aims to explore current strategies for reducing GVC following pancreatectomy, and seeks to identify effective interventions that minimize morbidity.

## Methods

### Protocol development

This systematic review was conducted in accordance with the Preferred Reporting Items for Systematic Reviews and Meta-Analyses (PRISMA) [5] guidelines, and was registered prospectively in the International Prospective Register of Systematic Reviews (PROSPERO) under the registration ID 635,692. A structured approach was adopted to identify and evaluate studies reporting on strategies to reduce gastric venous congestion (GVC) following pancreatic surgery.

The inclusion criteria were defined using the Population, Intervention, Comparison, Outcomes, and Study Design (PICOS) framework:

1. Population: patients undergoing pancreatectomy.
2. Interventions: surgical or perioperative strategies aimed at reducing GVC;
3. Comparators: standard care or alternative interventions;
4. Outcomes:
  - Primary outcome – incidence of gastric venous congestion.
  - Incidence of delayed gastric emptying (DGE), gastric ischaemia/necrosis, post pancreatectomy haemorrhahge (PPH), major morbidity (Clavien-Dindo ≥ 3, 90-day mortality, and length of stay.
5. Study Design: randomized controlled trials (RCTs), cohort studies, case-control studies, and case series/studies.

Studies were excluded if they were non-English language publications without translation or did not report outcomes relevant to GVC or its management.

### Search strategy, Data extraction and analysis

**Fig. 1:**
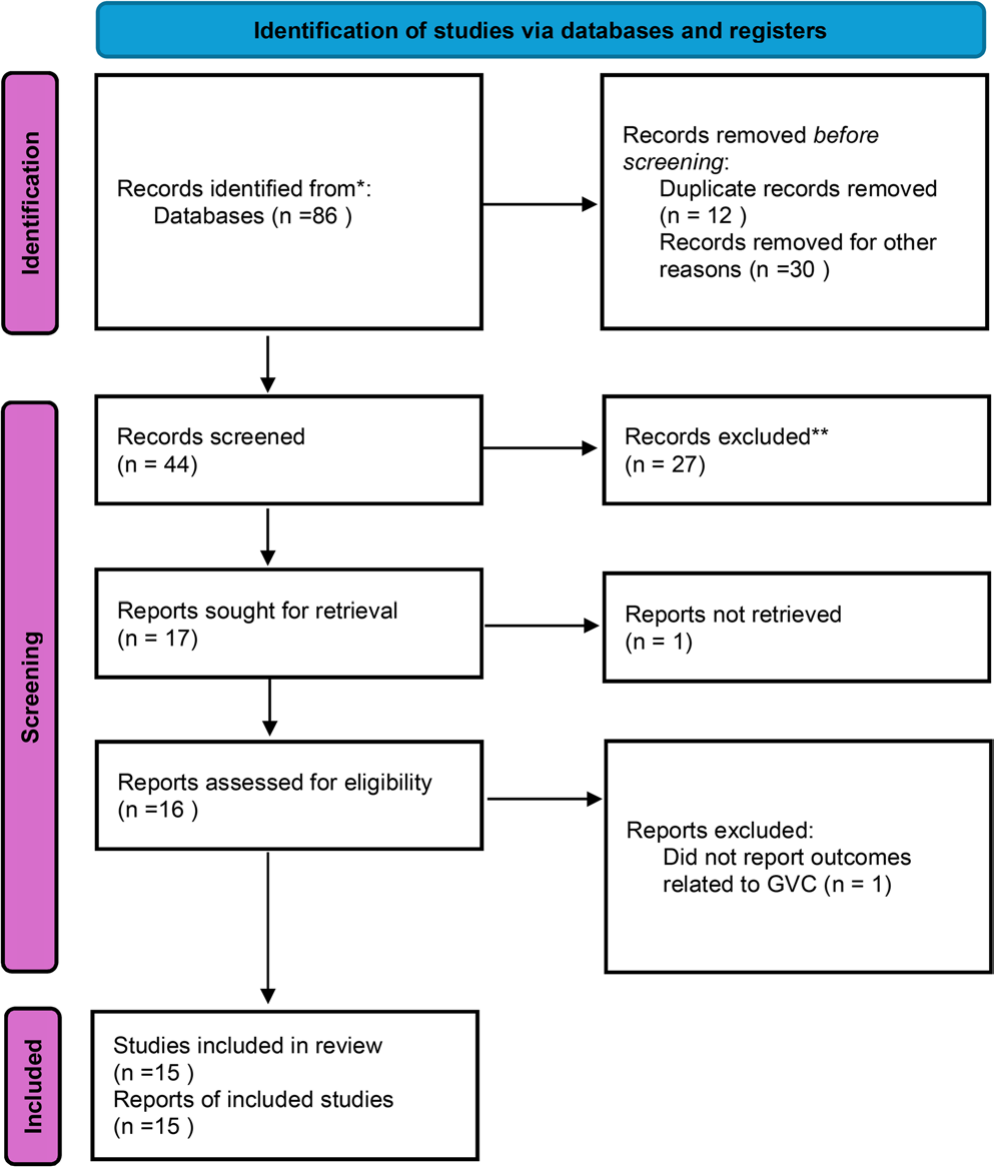
PRIMA flow diagram

While MEDLINE, Web of Science, and CENTRAL are recommended core databases, additional databases (Embase, Google Scholar) and grey literature were included to maximise sensitivity and capture surgical technical reports. Search terms used were (“Pancreaticoduodenectomy” OR “Pancreatoduodenectomy” OR “Whipple Procedure” OR “Pancreatic Surgery” OR “Total Pancreatectomy”) AND (“Gastric Venous Congestion” OR “Gastric Ischemia” OR “Venous Outflow Obstruction” OR “Hyperemia” OR “Venous Drainage”) (see Appendix 1: Search Strategy). Grey literature sources such as conference abstracts and clinical trial registries were also reviewed The PRISMA flow diagram (Fig. 1) was used to document the study selection process, detailing the number of records identified, screened, assessed for eligibility, and included in the final analysis.

Two independent reviewers (KK and HM) screened titles and abstracts for relevance before conducting full-text reviews of potentially eligible studies. Discrepancies were resolved through discussion or consultation with a third reviewer (KK2). Data extraction was performed using a standardized form to capture study characteristics, intervention details, outcomes, and quality assessment results. Risk of bias assessment was performed for retrospective cohort studies using the MINORS tool [6]. For case series and case reports, the Murad et al. [7]. tool for assessing case reports was used. After the individual studies were appraised the GRADE framework [8] was applied to assess the overall certainty of evidence derived from these reports. (Appendix 2, 3, and 4 for Risk of Bias assessments and GRADE assessment).

### Definitions

Pancreatic surgery-specific complications (i.e., DGE, PPH) were defined according to the ISGPS definitions, whereby only the clinically relevant grade B/C complications were registered. Major morbidity was defined as Clavien-Dindo grade IIIa or higher. Gastric venous congestion was defined as (1) congested gastric/perigastric veins with blue stomach discolouring, (2) gastric wall edema, (3) petechiae of the gastric serosa, and/or (4) congestion and/or hemorrhagic stomach mucosa as well as considerable venous bleeding when the stomach was opened [7]. 

### Quantitative synthesis

A single-arm meta-analysis of proportions was performed for outcomes reported in ≥ 5 studies with extractable numerator and denominator data. Small case series, Case reports and technical notes without denominators were excluded from quantitative synthesis. Outcomes analysed included intraoperative GVC, postoperative GVC, delayed gastric emptying, postpancreatectomy haemorrhage, major morbidity (Clavien–Dindo ≥ III), and 90-day mortality.

Pooled incidence estimates were calculated using a random-effects model with logit transformation to stabilise variance. A continuity correction (0.5) was applied for zero-event studies. Between-study heterogeneity was assessed using Cochran’s Q and quantified with the I² statistic.

Given substantial clinical and methodological heterogeneity across studies, analyses were restricted to descriptive incidence estimates. Subgroup analyses and publication bias assessment were not performed due to limited data. All analyses were conducted in Python (Google Colab).

## Results

### Study selection and characteristics

A total of 15 studies [1, 3–16] were published between 2005 and 2024 that investigated gastric venous congestion (GVC) following pancreatic surgery. The included studies comprised 6 retrospective cohort studies [1, 2, 11–15], 5 case reports [16–20], 2 case series [21, 22] and 2 technical notes [9, 23]. Sample sizes in the cohort studies ranged from 10 to 585 patients, with a median follow-up period ranging from hospital discharge to 35 months.

### Patient demographics and clinical characteristics

Data from a total of 1,133 patients were reported (Appendix 6: Patient and Clinical Characteristics). The median/mean age ranged from 49 to 74 years with a balanced gender distribution in most cohorts (43.1–56.9% female). The majority of studies did not report on patient comorbidities, with several reporting ASA class only. When reported, the most common comorbidities included cardiovascular disease (49.6% in one study), hypertension (41.4%), and diabetes mellitus (27.6%). The predominant surgical indication was pancreatic malignancy, primarily pancreatic ductal adenocarcinoma (PDAC). Other indications included intraductal papillary mucinous neoplasms (IPMN) (6.0–75%), neuroendocrine tumours (4.6–11%), chronic pancreatitis (1.8–6.8%), and other malignancies.

### Primary outcomes

#### Intraoperative GVC

**Fig. 2:**
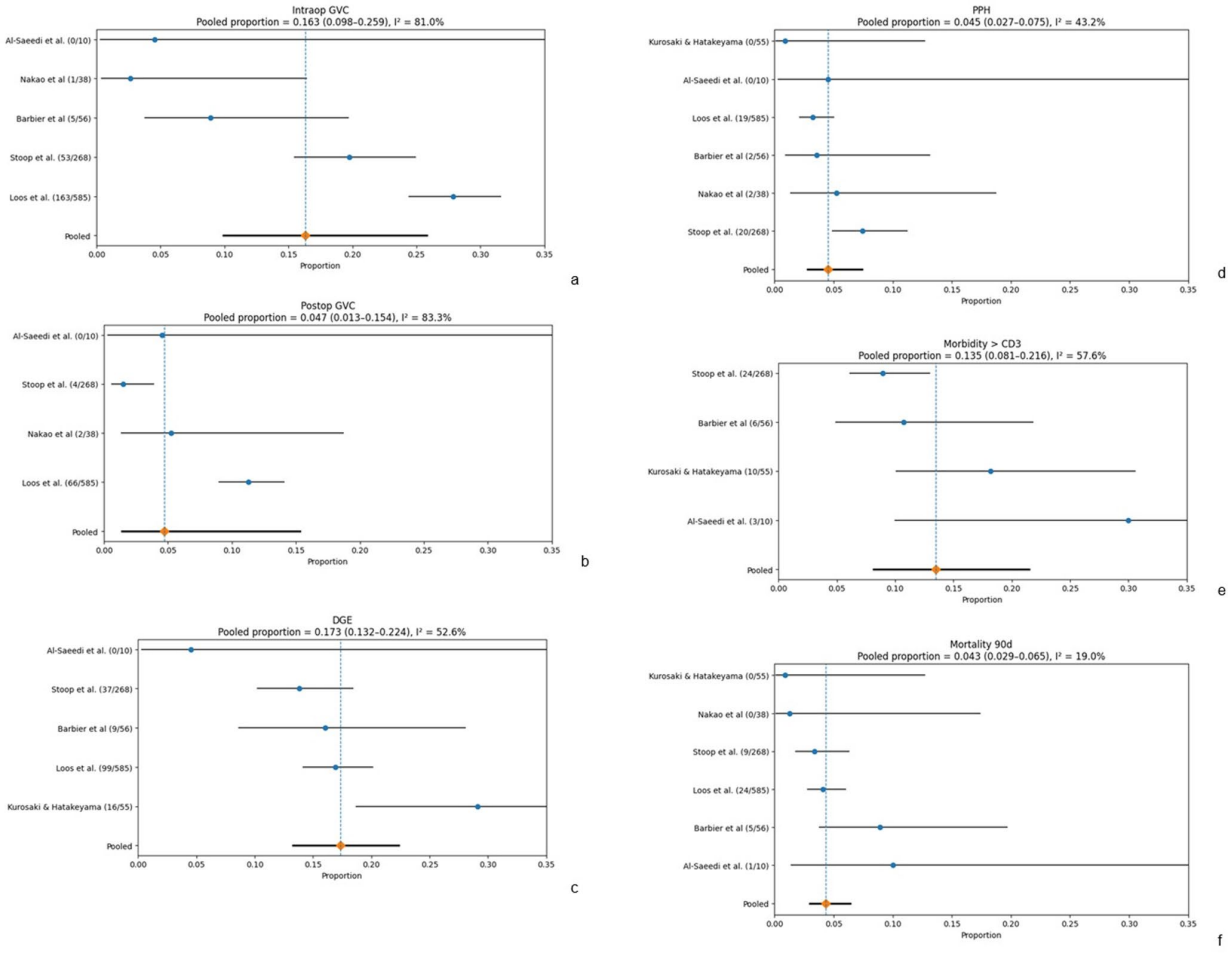
Single arm metanalysis (quantitative synthesis)

The incidence of intraoperative GVC was reported in 11 studies [1, 3, 11, 12, 15, 17–22], (See Table 1: Surgical Outcomes and Fig. 2: Intraoperative vs. post operative GVC) with incidence rates ranging from 5.0% to 27.9% in cohort studies. The highest rate was reported by Loos et al. [1], where 27.9% (163/585) of patients undergoing total pancreatectomy exhibited intraoperative GVC, characterized by gastric discoloration, venous engorgement, or edema. Similarly, Stoop et al. [3] documented intraoperative GVC in 21.3% (57/268) of cases. All case reports inherently featured intraoperative GVC due to their focus on severe or complex presentations. Diagnosis was primarily based on visual assessment, characterized by bluish discoloration, oedema, congestion, and visible venous engorgement. Notably, studies employing venous preservation or reconstruction strategies reported no postoperative GVC, suggesting the efficacy of proactive intraoperative management.

**Table 1:** Surgical and Vein management strategies

Author (Year)	Operation Type	Venous Resection Details	Gastric vein Preservation/Reconstruction Technique	GVC observed Intraoperatively (%)	Strategies to manage GVC when observed intraoperatively
Kurosaki (2005) [14]	PPPD (100%)	PV resection in 3.6% (2/55) as additional procedure	LGV preservation in 40% (22/55)	NR	Antecolic reconstruction with stomach set vertically
Sandroussi (2010) [17]	TP with splenectomy	PV-SMV segment resection (100%) with end-to-end anastomosis	LGV to IMV anastomosis (100%)	Yes	LGV-IMV bypass with running suture
Barbier (2013) [15]	TP	Gastric venous resection in 11% (6/56), no PMV axis resection	LGV preservation in 5.4% (3/56)	9% (5/56)	Pylorus preservation (57%), spleen preservation (20%)
Hackert (2015) [9]	PD with PVR	PV resection with particular focus on LGV-PV junction location	LGV reinsertion to PV (end-to-side) with potential SV patch	Yes	LGV-PV end-to-side anastomosis with running sutures
Nakao (2018) [12]	TP variants including TPDG (42.1%)	Portomesenteric venous resection in 63.2% (24/38) with catheter-bypass used in 54.2% (13/24)	LGV preserved in 39.5% (15/38); RGEV-ovarian vein anastomosis in 2.6% (1/38)	NR	Distal gastrectomy (42.1%), venous preservation where possible
Strobel (2018) [23]	Extended pancreatectomy	Portomesenteric venous confluence resection	Splenic vein to left renal vein shunt	Yes	Distal splenorenal shunt technique
Kagota (2020) [19]	Remnant TP	Splenic vein resection	LGV to SpV end-to-end anastomosis using 6 − 0 nylon	Yes	LGV-SpV anastomosis
Shiihara (2020) [11]	PD with PVR	PV-SMV reconstruction using 5 − 0/6 − 0 non-absorbable running suture; SPV ligated in 33.3% (36/108), LGV ligated in 25% (23/92)	Preservation of IMV and LGV attempted “to greatest extent possible”	NR	Venous preservation strategy where possible
Al-Saeedi (2021) [13]	PD (60%), TP (40%)	Portal venous confluence resection (100%) with end-to-end SMV-PV anastomosis	Splenic vein reimplantation via splenorenal shunt (6 − 0 monofilament)	Yes	Splenorenal shunt with Doppler flowmetry assessment
Loos (2022) [7]	TP (87.9%), completion TP (12.1%)	Portal/SMV resections in 37.1% (217/585), left gastric vein resection in 13.8% (81/585)	Not detailed	27.9% (163/585)	Partial gastrectomy (26.3%, 154/585)
Kokoroskos (2023) [18]	TP with splenectomy	Left gastric vein and splenic vein sacrificed during dissection	None attempted	No	None during index operation; reoperation with gastrectomy
Nakamura (2023) [21]	TP variants	Not specified	LGV preserved (60%, 3/5), PGV/SGV/SPV preserved (20%, 1/5)	No	Tailored gastrectomy extent based on vein preservation
Stoop (2023) [1]	TP (86.6%), completion TP (13.4%)	Portomesenteric venous resection in 34.7% (93/268); segmental venous resection (types 3–4) in 30.2% (81/268)	Left gastric vein ligated in 21.6% (58/268), splenic vein ligated in 86.9% (233/268)	21.3% (57/268)	Gastrectomy in 51% of GVC cases (29/57)
Fernández-Placencia (2024) [20]	RAMPS	LGV and splenic vein resection (100%) for oncologic reasons	LGV to left adrenal vein anastomosis using 6 − 0 polypropylene	100%	LGV-LAV anastomosis (running posterior, interrupted anterior)
Reddy (2024) [22]	TP variants	Multiple approaches: Case 1: Venous involvement requiring reimplantation; Case 2: 3 cm segmental PV resection; Case 3: Venous conduit construction	Case 1: LGV-IMV + RGEV-colic vein; Case 2: PV end-to-end; Case 3: LGV-RGEV conduit to MPV	100%	Microvascular reconstruction techniques
Yamanaka (2024) [16]	SSPTP → TG	LGV and SpA transection without formal venous resection/reconstruction	None	100%	Total gastrectomy when congestion persisted

#### Postoperative GVC

Postoperative GVC was less frequently reported, in eight studies [1, 2, 11, 17, 18, 20–22], but carried substantial morbidity. Shiihara et al. [11] observed postoperative varices (a surrogate marker for GVC) in 24.1% (26/108) of patients after pancreatoduodenectomy with portal vein resection. Loos et al. [1] identified 5.0% (29/585) of patients requiring relaparotomy for postoperative GVC, diagnosed via CT findings (pneumatosis, free gas) or endoscopic evidence of mucosal necrosis.

#### Surgical Interventions and venous management strategies

From the 16 included studies, a total of 1,132 procedures were performed (see Table 2). Total pancreatectomy (TP) variants were most common, in 960 cases (84.8%). The second most common procedure was pancreatoduodenectomy (PD) variants in approximately 170 patients (15%), including standard PD, pylorus-preserving PD, and PD with portal vein resection. Other procedures such as RAMPS and remnant pancreas resection were performed in only 2 patients. Venous resection rates varied considerably with major portal/superior mesenteric vein resection performed in 217 cases from the Loos et al. cohort alone. Venous preservation or reconstruction strategies showed significant heterogeneity, including LGV preservation (22–60% where reported), splenic vein preservation, and various venous anastomoses. Intraoperative gastric venous congestion was reported in 21–28% of patients in the larger cohorts, with partial or total gastrectomy performed in 26.3% of cases in the Loos et al. series and 10.8% in the Stoop et al. series when congestion was detected.

**Table 2:** Data used for quantitative synthesis

Author	Year	Study Design	Study size	TP	Splenectomy - TP	PD	DP	Splenectomy - DP	Pylorus preservation	Partial gastrectomy	PV-SMV resection	Arterial Resection	LGV resection	Intraop GVC	Postop GVC	DGE	PPH	GJ leak	Reoperation for GVC	Morbidity > CD3	Mortality 90d
Loos et al.	2022	Retrospective cohort study	585	585	426	0	0	N/A	187	62	217	67	31	163	66	99	19	14	120	NR	24
Stoop et al.	2023	Retrospective observational single-center study	268	268	233	N/A	N/A	N/A	90	29	93	25	58	53	4	37	20	3	18	24	9
Nakao et al.	2018	Retrospective Cohort study	38	38	38	0	0	N/A	17	24	24	0	25	1	2	NR	2	NR	1	NR	0
Kurosaki & Hatakeyama	2005	Retrospective Cohort study	55	0	N/A	55	N/A	N/A	55	0	2	0	33	N/A	N/A	16	0	0	0	10	0
Barbier et al.	2013	Retrospective cohort study with prospective QoL assessment	56	56	45	0	0	N/A	32	1	N/A	N/A	3	5	N/A	9	2	2	2	6	5
Al-Saeedi et al.	2021	Retrospective observational study	10	4	0	6	0	N/A	10	0	10	4	N/A	0	0	0	0	0	0	3	1

TP – total Pancreatectomy

PD – pancreatoduodenectomy

DP – Distal pancreatectomy

PV-SMV – portal vein-superior mesenteric vein

LGV – left gastric vein

GVC – gastric venous congestion

DGE – delayed gastric emptying

PPH – post pancreatectomy haemorrhage

GJ – gastrojejunostomy/duodenojejunostomy

CD – Clavien-Dindo

### Secondary outcomes

#### Post operative mortality

Six studies reported 90 day mortality rates ranging from 0% to 10%, with higher rates observed in patients with GVC (Table 2: Clinical Outcomes). Loos et al. reported 90-day mortality of 7.4% in GVC patients versus 2.8% in non-GVC patients (*p* = 0.014). Mean/median length of stay ranged from 7 to 27.2 days, with significantly longer hospitalization for patients with GVC in comparative studies (19 vs. 16 days, *p* < 0.001 in Loos et al.).

#### Delayed gastric emptying (DGE)

DGE rates were reported in six studies [1, 2, 13, 15, 20, 22]. Barbier et al.¹³ reported DGE in 16% of patients after total pancreatectomy, while Stoop et al.³ documented 13.9% (37/268) in their cohort. Case series employing venous reconstruction techniques, such as LGV-to-splenic vein anastomosis¹⁷, noted no DGE, underscoring the potential role of preserved venous drainage in maintaining gastric motility.

#### Gastric Ischemia/Necrosis

Gastric ischemia or necrosis rates were reported in 5 studies [2, 17, 18, 20, 22], and was observed in 5.0% (29/585) of patients in the Loos et al.³ cohort, primarily in cases where GVC was unrecognized intraoperatively. Kokoroskos et al.¹⁶ described a catastrophic case requiring emergency total gastrectomy due to irreversible ischemia from unaddressed GVC.

#### Postpancreatectomy hemorrhage (PPH) and Pancreatic fistula (POPF)

PPH was only reported in two studies [2, 15], with a rate of 5.8% (34/585) of patients by Loos et al.³, often associated with vascular resection. POPF was also reported in only two studies [14, 15], with a incidence of 9.1% (5/55) of cases in Kurosaki & Hatakeyama’s cohort¹². Neither complication was directly linked to GVC in most studies, though their coexistence may exacerbate morbidity.

#### Major complications (Clavien-Dindo ≥3a)

Major complications were frequent in high-risk cohorts, reported in four studies [1, 11, 13, 21]. Stoop et al.³ reported a 28.0% (75/268) rate of Clavien-Dindo ≥IIIa events, including sepsis and organ failure, while Al-Saeedi et al.¹¹ noted 30% (3/10) major complications despite successful splenorenal shunts.

#### Mortality

Five studies [1, 2, 13, 20, 22] reported 90 day mortality, ranging from 0% in case reports¹⁵,¹⁷ to 10% in Al-Saeedi et al.¹¹. Loos et al.³ highlighted significantly higher mortality in GVC patients (7.4% vs. 2.8%, *p* = 0.014), emphasizing the critical need for early recognition and intervention.

#### Length of stay

Seven studies [1, 2, 13, 18, 20–22] reported on length of hospital stay, which were shown by Loos et al. to be prolonged in GVC patients, with a median of 19 days vs. 16 days in non-GVC patients (*p* < 0.001)³. Extended stays correlated with reoperations, infections, or nutritional challenges following gastrectomy.

### Risk Factors for gastric venous congestion

Multivariate analyses from the larger cohort studies identified several independent risk factors for GVC. These were;

1. Left gastric vein ligation: OR for GVC of 5.49 (*p* < 0.001) in Loos et al.; and OR of 11.86 (*p* < 0.001) in Stoop et al.
2. Splenectomy: OR for GVC of 2.14 (*p* = 0.007) in Loos et al.
3. Portal vein resection: OR for GVC of 2.10 (*p* = 0.040) in Stoop et al.
4. Combined vascular resections: The highest risk was observed when multiple venous drainage pathways were sacrificed.

### Quantitative synthesis (single arm metanalysis)

Quantitative synthesis was feasible for selected incidence outcomes reported in ≥ 3 studies with extractable numerator and denominator data. Given anticipated clinical and methodological heterogeneity, pooled estimates are presented as descriptive.

The pooled incidence of intraoperative gastric venous congestion (GVC) was 16.3% (95% CI 9.8–25.9; I²=81.0%), while postoperative GVC occurred in 4.7% of cases (95% CI 1.3–15.4; I²=83.3%). Delayed gastric emptying (DGE) was observed in 17.3% of patients (95% CI 13.2–22.4; I²=52.6%). The pooled incidence of postpancreatectomy haemorrhage (PPH) was 4.5% (95% CI 2.7–7.5; I²=43.2%), and major morbidity (Clavien–Dindo ≥ III) occurred in 13.5% (95% CI 8.1–21.6; I²=57.6%). Ninety-day mortality was 4.3% (95% CI 2.9–6.5; I²=19.0%) (Fig. 2a *– f*).

Heterogeneity was substantial for intraoperative and postoperative GVC, likely reflecting variation in diagnostic definitions, intraoperative assessment, and procedure type across studies. In contrast, mortality demonstrated low heterogeneity, suggesting greater consistency in this outcome. These findings provide a quantitative summary of reported event rates but should be interpreted cautiously given the underlying heterogeneity and predominance of retrospective data.

### Management strategies and outcomes

Preservation or reimplantation of venous drainage pathways represented a key focus across studies (see appendix 7). LGV preservation was reported in 34.2–60% of cases where documented. Various reimplantation techniques were described; these included.

1. LGV to inferior mesenteric vein (IMV) anastomosis [17].
2. LGV to splenic vein anastomosis [19].
3. LGV to left adrenal vein anastomosis [20].
4. Distal spleno-renal shunts (splenic vein to left renal vein) [13, 23].
5. End-to-side left gastric vein reimplantation to portal vein [9].

All cases of reconstruction reported no postoperative gastric venous congestion, or associated effects (e.g. delayed gastric emptying). When venous reconstruction was not feasible, partial or total gastrectomy was performed. Rates varied widely, from 1.8% [15] to 42.1% [12]. In the largest series, Loos et al. reported gastrectomy for intraoperative GVC in 26.3% (154/585) of patients.

## Discussion

GVC is a critical driver of adverse outcomes following pancreatectomy, with intraoperative incidence exceeding 20% in major cohort studies of high risk patients³. Sequelae of GVC, such as DGE and ischemia, further contribute to morbidity. Standardized protocols for venous assessment and reconstruction may mitigate these risks, as evidenced in the case reports employing such strategies¹⁷⁻¹⁹. Heterogeneity in outcome reporting, however, underscores the need for consensus definitions to enable comparative analyses.

### Anatomical considerations and pathophysiology

The development of GVC following pancreatic resection fundamentally stems from disruption of the venous drainage system of the stomach. Multiple studies in our review highlighted the anatomical variations that influence GVC risk. Hackert et al. [4] described two predominant anatomical variants: Type 1, where the LGV drains directly into the portal vein, and Type 2, where it drains into the splenic vein. This distinction carries significant implications for surgical planning, as Type 2 variants are more susceptible to venous outflow compromise during splenic vein resection. Other variant anatomy may also affect outcomes; Yamanaka et al. [16] detail a case of a replaced common hepatic artery branching from the superior mesenteric artery, leading to hemodynamic changes after splenic artery transection and subsequent GVC.

Procedure type appears to play an important role in the development of gastric venous congestion (GVC), reflecting the differing patterns of venous disruption between right- and left-sided pancreatic resections. In pancreatoduodenectomy, venous drainage of the stomach is typically preserved unless combined with portal or superior mesenteric vein resection involving the left gastric vein. In contrast, distal pancreatectomy—particularly when combined with splenectomy—directly disrupts the splenic vein, short gastric veins, and left gastroepiploic vein, which are critical components of gastric venous outflow. This can lead to sinistral (left-sided) portal hypertension, characterised by elevated pressure within the splenic venous system and the development of gastric varices. While clinically overt GVC may be less frequently reported following distal pancreatectomy, likely due to under-recognition or differing definitions, the underlying haemodynamic disturbance is well established and may manifest as delayed gastric emptying, variceal formation, or gastrointestinal bleeding. These differences highlight the importance of procedure-specific risk assessment and reinforce the need to consider venous preservation strategies, particularly in the setting of splenic vein ligation. Several studies also note the concept of sinistral (left sided) portal hypertension as a contributing mechanism to GVC. Shiihara et al. [11]. described the process of portal or splenic vein obstruction, with subsequent sinistral portal hypertension leading to GVC when alternative collateral pathways are simultaneously sacrificed. They observed a 60% incidence of left-sided varices when all splenic venous drainage pathways were compromised, compared to only 16.7% when at least one pathway was preserved. Fernández-Placencia et al. [20] similarly documented severe GVC resulting from a combination of tumour infiltration of the LGV and splenic vein with resulting sinistral portal hypertension. Splenomegaly and sequestration (in patients with splenic preservation) subsequent to the sinistral portal hypertension may also result in delays in administration of adjuvant chemotherapy.

### Preoperative assessment and planning

Preoperative evaluation of venous anatomy has emerged as a critical step in GVC prevention, with CT angiography facilitating mapping of gastric venous drainage patterns and appropriate surgical planning. Preservation of key venous drainage pathways, when oncologically feasible, was consistently recommended across all included studies in this review (Appendix 7). Shiihara et al. [11] proposed a hierarchical approach to venous preservation: primarily maintaining splenic venous drainage when possible, but if splenic vein ligation is unavoidable, preserving alternative drainage paths such as the left gastric vein via portal vein trunk or inferior mesenteric vein via splenic vein. Several studies also noted that pylorus preservation combined with right gastric venous preservation provides an additional venous drainage pathway.

### Venous reconstruction strategies

When preservation of native venous drainage is impossible, reconstruction represents an alternative strategy that may reduce the need for gastrectomy. However, a systematic strategy towards venous reconstruction does not yet exist. Our systematic review identified multiple innovative techniques across the included studies, each with specific technical considerations and applications.

**Fig. 3:**
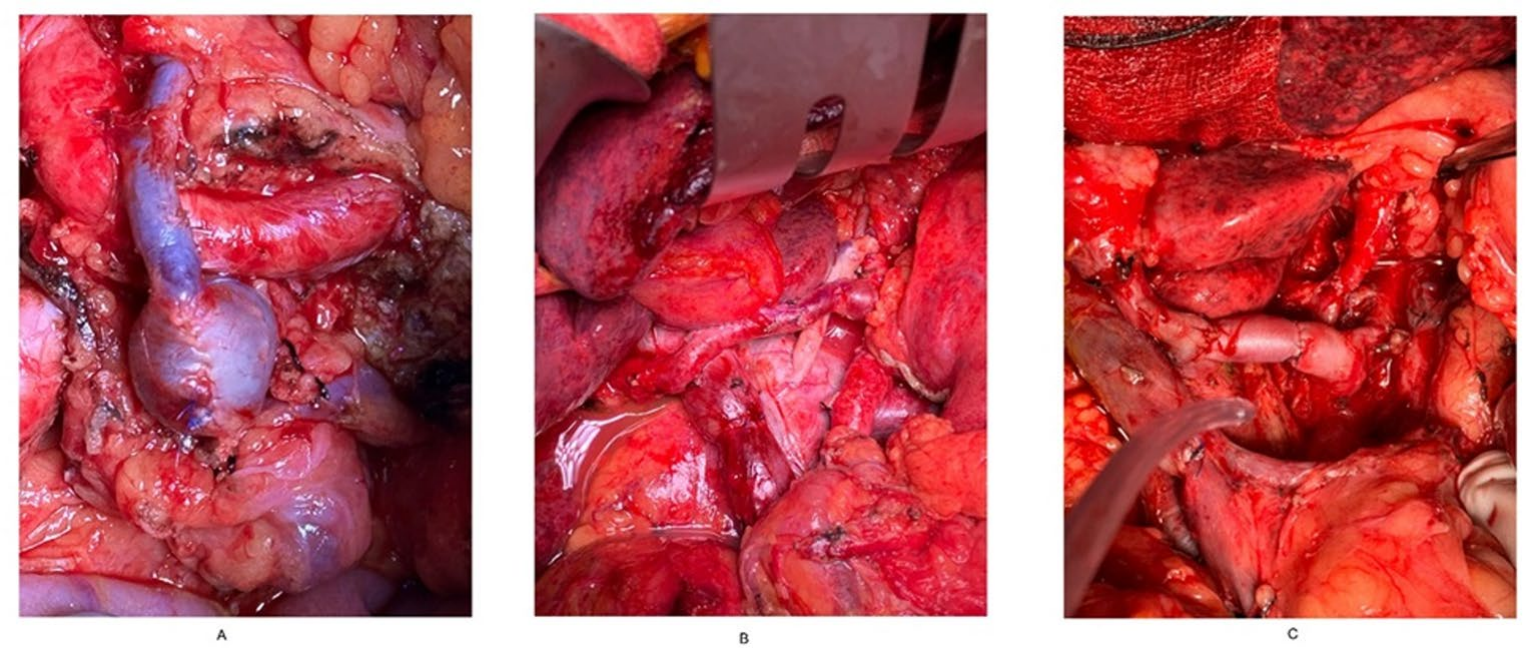
**a**. Left gastric vein into splenic vein anastomosis. **b**. Left gastric vein to left renal vein anastomosis. **c**. Left gastric vein to dilated umbilical vein anastomosis

Hackert et al. [4] provided a detailed technical description of end-to-side left gastric vein reinsertion to the portal vein, potentially using a splenic vein patch for larger anastomosis. This technique is particularly applicable for Type 1 left gastric vein anatomy when portal vein resection is required. The authors emphasized several technical nuances, including flushing the left gastric vein and tying sutures under perfusion to avoid a purse-string effect, while preventing left gastric vein twisting during anastomosis. They also recommended an flow assessment to ensure anastomosis patency – this can be performed with intraoperative doppler. When direct reimplantation to the portal vein is not feasible, alternative bypass routes have been described. Sandroussi and McGilvray reported successful LGV to IMV bypass in a case of severe GVC following total pancreatectomy with portal vein resection. The authors emphasized the importance of preserving the IMV in continuity with the portal vein to allow it to function as an outflow pathway. Similarly, after observing intraoperative GVC, Kagota et al. performed an end-to-end anastomosis between the LGV and splenic vein, with immediate resolution of gastric congestion. This technique has also been performed successfully in our centre (Fig. 3). Fernández-Placencia et al. described a novel technique involving LGV to left adrenal vein anastomosis (running 6 − 0 polypropylene on posterior surface, interrupted on anterior surface), which may be limited by the calibre of the adrenal vein. For cases with portal confluence resection, Strobel et al. and Al-Saeedi et al. described a distal spleno-renal shunt technique, creating an end-to-side anastomosis between the splenic vein and left renal vein. Al-Saeedi et al. emphasized that this procedure is technically straightforward, requiring only 5–15 min of additional operative time, and intraoperative Doppler flowmetry. In their series of 10 patients, all shunts remained patent at follow-up, with no instances of postoperative GVC. We recommend dividing the splenic vein only after dissecting a considerable length of vein away from the pancreas– dissection and isolation of the splenic vein following vein division is challenging due to engorgement and hypertension.

Reddy et al. describe more complex scenarios requiring multiple venous reconstructions. In one case, they performed dual venous reconstruction (LGV to IMV and right gastroepiploic vein to colic vein) to provide redundancy against thrombosis of a single reconstructed vein. In another, they created an LGV-RGEV conduit to the main portal vein. The authors recommended intraoperative “bulldog clamp tests” before permanent vessel ligation to evaluate the impact on gastric perfusion and emphasized the potential benefit of microvascular surgical expertise for these complex reconstructions. In our centre, successful LGV to left renal vein anastomosis (*Image 2*) and LGV into a re-dilated umbilical vein have also been performed.

Common to all reconstruction techniques, careful attention to technical details is emphasized. Many authors recommended using fine (6 − 0 or smaller) non-absorbable monofilament, tension-free anastomoses, and confirming flow with intraoperative Doppler assessment. The consistent absence of postoperative GVC following these reconstructions suggests their efficacy in maintaining adequate gastric venous drainage.

When venous preservation or reconstruction is not feasible, several studies recommended adjusting the extent of gastrectomy based on the degree of venous compromise. However, it should be noted that gastrectomy has potential long-term nutritional implications, particularly when combined with the exocrine and endocrine insufficiency that follows pancreatic resection.

### Proposed approach for management of GVC in Pancreatic surgery

**Fig. 4:**
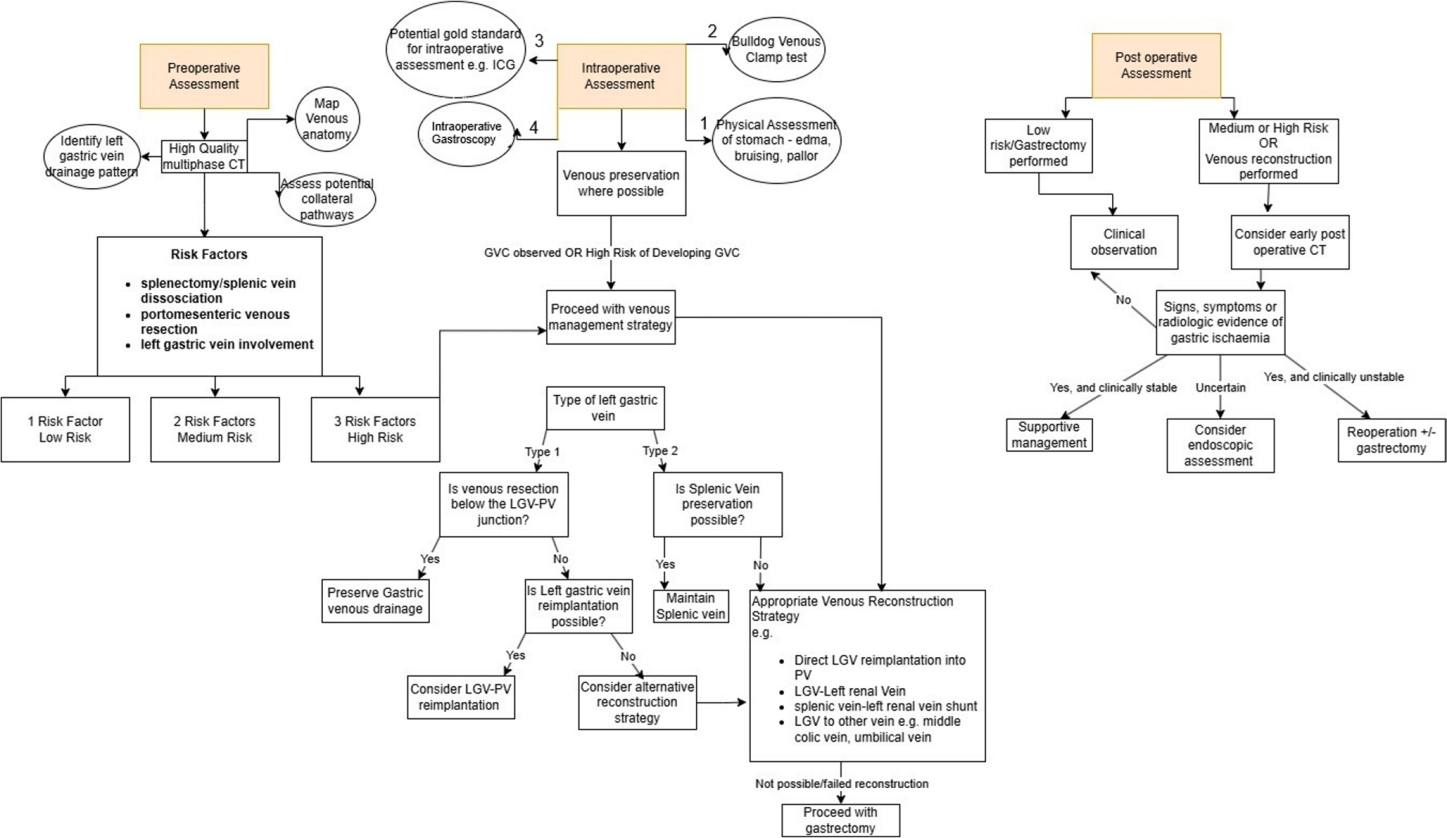
Suggested protocol for standardised approach to gastric venous congestion

Although the above data is considerable heterogenous, we propose the following preliminary approach for decision-making regarding venous management during pancreatic surgery (Fig. 4: Algorithm for proposed approach);

#### Preoperative assessment

1. High-quality multiphasic CT with 3D vascular reconstruction.

- Map portal, splenic, and mesenteric venous anatomy.
- Identify left gastric vein drainage pattern (Type 1: LGV drains into PV; Type 2: LGV drains into SpV).
- Assess potential collateral pathways (IMV, renal vein).
- Evaluate relationship between tumour and key venous structures.

2. Risk stratification.

- High risk of GVC: Planned splenectomy, portal vein resection and left gastric vein involvement.
- Moderate risk of GVC: Two risk factors present.
- Low risk of GVC: Single risk factor or no venous involvement.

3. Potential involvement of microvascular surgeons for reconstruction.

#### Intraoperative decision algorithm

1. Initial Assessment.

- If the tumor does not involve major venous structures, then aim to preserve all major gastric drainage veins.
- If venous involvement is present, then proceed to step 2 (Venous management strategy).

2. Venous Management Strategy.

- Type 1 Left Gastric vein.
  - If PV resection is performed below the LGV-PV junction, then Preserve LGV drainage.
  - If PV resection includes the LGV-PV junction, then consider LGV reimplantation back into the PV.
  - If LGV reimplantation is not feasible, assess alternative reconstruction options.
  - Type 2 Left Gastric Vein.
    - If splenic preservation is possible, then maintain SpV-PV continuity.
    - If splenectomy is required, then consider LGV reimplantation into the PV or an alternative bypass.
  3. Intraoperative GVC Assessment.
- A simple clamp test must be performed prior to permanent vessel ligation, looking for signs of GVC, which include gastric discoloration, oedema, visible venous engorgement.
- Standardised indocyanine Green assessment of stomach wall for perfusion assessment may be conducted.
- If GVC is observed in either of these, then implement the reconstruction strategy or proceed to gastrectomy.
- Intraoperative gastroscopy may provide direct mucosal assessment, allowing early detection of venous congestion or ischemia not apparent on serosal inspection. It may be considered as an adjunct during intraoperative assessment.

4. Options for Reconstruction Options include;
  1. Direct LGV reimplantation to the PV.
  2. LGV to left renal vein (LRV) anastomosis.
  3. Splenic vein to LRV shunt (if splenic vein available).
  4. LGV to other available venous structures (colic vein, IMV, umbilical vein, ovarian vein etc.)
5. Gastrectomy Decision.

- If no venous reconstruction options available and GVC is present:
  - If venous preservation was not possible, consider subtotal/total gastrectomy.
  - If only partial venous preservation was possible, consider distal gastrectomy.
  - If reconstruction has failed or is inadequate, with persistent GVC, then proceed to appropriate gastrectomy.

#### Postoperative monitoring

1. If the patient is high risk, consider early postoperative CT angiography (day 5–7) to assess:

- Venous reconstruction patency.
- Signs of gastric wall oedema or ischemia.
- Continue close monitoring for symptoms.

2. Consider endoscopic assessment if signs and symptoms of GVC present/clinical suspicion of GVC:

- Persistent nausea/vomiting, high NG output, inadequate oral intake, and abdominal distension suggestive of DGE.
- Hematemesis or melena, severe epigastric pain, hemodynamic compromise, inflammatory marker rise or lactatemia suggestive of gastric ischemia or necrosis.

3. Reoperation for confirmed postoperative GVC with:

- Attempted venous reconstruction if technically feasible.
- Partial/total gastrectomy if reconstruction not possible.

## Limitations

Most included studies were retrospective cohort studies, while five were single case reports and two were technical notes without patient outcome data. Methodological quality was generally moderate, with MINORS scores ranging from 10 to 16/24 for cohort studies. Significant heterogeneity existed across studies in operative techniques, definitions of gastric venous congestion, follow-up duration, and outcome measures. Additionally, publication bias likely favoured reporting of successful venous reconstruction techniques over unsuccessful attempts.

There is no standardized diagnostic criteria for GVC, relying primarily on subjective visual assessment rather than objective measures like ICG fluorescence. Development of standardized definitions of GVC assessment and reconstruction would then allow for the development and validation of risk prediction tools could help identify patients who might benefit from prophylactic venous reconstruction or modified surgical approaches.

The technical notes and case reports included in this review, while providing valuable insights into innovative reconstruction techniques, represent limited experience that may not be reproducible in all settings. The feasibility of complex venous reconstructions is likely dependent on surgeon experience, institutional resources, and patient-specific factors not fully captured. Finally, comparative studies of different reconstruction techniques are needed to determine the optimal approach for specific anatomical scenarios.

An additional limitation relates to the quantitative synthesis. The meta-analysis was restricted to single-arm pooled incidence estimates derived from a small number of heterogeneous, predominantly retrospective studies. Variability in definitions of GVC, differences in operative approach and case mix, and inconsistent outcome reporting limit the precision and generalisability of the pooled estimates. Furthermore, the lack of procedure-specific and risk-stratified data precluded meaningful subgroup analyses, and the small number of studies per outcome limited assessment of publication bias.

## Conclusion

This systematic review demonstrates that GVC represents a significant complication following pancreatic resection, particularly total pancreatectomy, or cases requiring venous resection. A multimodal approach to prevention – incorporating detailed preoperative planning, selective venous preservation, innovative reconstruction techniques, and judicious use of gastrectomy – appears most effective in mitigating this risk. The proposed protocol provides a structured approach to decision-making that may help reduce the incidence and impact of this complication. Future research should focus on standardizing GVC assessment techniques. Various venous reconstruction approaches should be compared, as well as an algorithm-based decision-making tool to optimize venous management during complex pancreatic resections.

## Supplementary Information

Below is the link to the electronic supplementary material.

Supplementary Material 1 (DOCX 13.7 KB)

Supplementary Material 2 (DOCX 15.7 KB)

Supplementary Material 3 (DOCX 16.3 KB)

Supplementary Material 4 (DOCX 16.2 KB)

Supplementary Material 5 (DOCX 16.8 KB)

Supplementary Material 6 (DOCX 27.6 KB)

Supplementary Material 7 (DOCX 17.5 KB)

## Data Availability

No datasets were generated or analysed during the current study.
