# Supplementary material for: Gastric venous congestion after pancreatic surgery: A systematic review, metanalysis and suggested protocol for assessment and management
Source: Langenbecks Arch Surg. 2026 Apr 25;411(1):159. doi: 10.1007/s00423-026-04049-8 (PMC13249684; doi:10.1007/s00423-026-04049-8)
Supplement: Supplementary file 1 — Supplementary Material 1 (DOCX 13.7 KB) [file 423_2026_4049_MOESM1_ESM.docx]

**Appendix 1: Search Strategy**

("Pancreaticoduodenectomy" OR "Pancreatoduodenectomy" OR "Whipple Procedure" OR "Pancreatic Surgery" OR “Total Pancreatectomy”)

AND

("Gastric Venous Congestion" OR "Gastric Ischemia" OR "Venous Outflow Obstruction" OR "Hyperemia" OR "Venous Drainage")

**Filters to Apply**

- Language: English

- Publication Date: No limit initially; refine based on relevance.

- Study Types: ALl

**Search Refinement**

- Use filters for human studies only.

- Use citation tracking to identify additional relevant articles from reference lists of key studies.
