# Supplementary material for: Gastric venous congestion after pancreatic surgery: A systematic review, metanalysis and suggested protocol for assessment and management
Source: Langenbecks Arch Surg. 2026 Apr 25;411(1):159. doi: 10.1007/s00423-026-04049-8 (PMC13249684; doi:10.1007/s00423-026-04049-8)
Supplement: Supplementary file 2 — Supplementary Material 2 (DOCX 15.7 KB) [file 423_2026_4049_MOESM2_ESM.docx]

**Appendix 5: Study and Center**

| **Author (Year)** | **Study Design** | **Country** | **Center Type** | **Sample Size** | **Follow-up Duration** |
| --- | --- | --- | --- | --- | --- |
| Kurosaki (2005) | Retrospective cohort | Japan | Single (Niigata University) | 55 | Until discharge |
| Sandroussi (2010) | Case report | Canada | Single (Toronto General Hospital) | 1 | 10 days |
| Barbier (2013) | Retrospective cohort with prospective QoL | France | Single (Hôpital Beaujon) | 56 | Median 35 months [4-168] |
| Hackert (2015) | Technical note | Germany | Single | N/A | N/A |
| Nakao (2018) | Retrospective cohort | Japan | Two centers (Nagoya University and Nagoya Central Hospital) | 38 | Until discharge |
| Strobel (2018) | Technical note | Germany | Single (Heidelberg University Hospital) | N/A | N/A |
| Kagota (2020) | Case report | Japan | Single (Osaka Medical College) | 1 | 22 months |
| Shiihara (2020) | Retrospective cohort | Japan | Single (Tokyo Women's Medical University) | 108 | Median 34.5 months |
| Al-Saeedi (2021) | Retrospective cohort | Germany | Single (Heidelberg University Hospital) | 10 | ≥13 months |
| Loos (2022) | Retrospective cohort | Germany | Single (Heidelberg University Hospital) | 585 | 90 days |
| Kokoroskos (2023) | Case report | Greece | Single (Konstantopouleio General Hospital) | 1 | 7 days |
| Nakamura (2023) | Case series | Japan | Single (Showa University Fujigaoka Hospital) | 5 | 6 months |
| Stoop (2023) | Retrospective cohort | Sweden | Single (Karolinska University Hospital) | 268 | 90 days |
| Fernández-Placencia (2024) | Case report | Peru | Single (Instituto Nacional de Enfermedades Neoplásicas) | 1 | 12 months |
| Reddy (2024) | Case series | India | Single (Tata Memorial Centre, Mumbai) | 3 | 6-64 months |
| Yamanaka (2024) | Case report | Japan | Single (Isesaki Municipal Hospital) | 1 | 18 months |
