# Supplementary material for: Gastric venous congestion after pancreatic surgery: A systematic review, metanalysis and suggested protocol for assessment and management
Source: Langenbecks Arch Surg. 2026 Apr 25;411(1):159. doi: 10.1007/s00423-026-04049-8 (PMC13249684; doi:10.1007/s00423-026-04049-8)
Supplement: Supplementary file 3 — Supplementary Material 3 (DOCX 16.3 KB) [file 423_2026_4049_MOESM3_ESM.docx]

**Appendix 2: Study Quality Assessment for cohort studies**

| **Author** | **Study type** | **Clear aim** | **Consecutive patients** | **Prospective data collection** | **Appropriate endpoints** | **Unbiased endpoint assessment** | **Appropriate follow-up interval** | **Loss to follow-up less than 5%** | **Prospective study size** | **Adequate control** | **Contemporary groups** | **Baseline Equivalence** | **Appropriate Statistics** | **Total** |
| --- | --- | --- | --- | --- | --- | --- | --- | --- | --- | --- | --- | --- | --- | --- |
|  | | /2 | /2 | /2 | /2 | /2 | /2 | /2 | /2 | /2 | /2 | /2 | /2 | /24 |
| Loos et al | Retrospective cohort study | 2 | 2 | 2 | 2 | 2 | 2 | 1 | 2 | 0 | 0 | 0 | 2 | 17 |
| Stoop et al | Retrospective cohort study | 2 | 2 | 1 | 2 | 2 | 2 | 1 | 0 | 0 | 2 | 1 | 2 | 17 |
| Shiihara et al | Retrospective cohort study | 2 | 2 | 0 | 2 | 1 | 2 | 0 | 0 | 0 | 2 | 1 | 2 | 14 |
| Nakao Et al | Retrospective cohort study | 2 | 1 | 0 | 2 | 1 | 2 | 1 | 0 | 0 | 1 | 0 | 0 | 10 |
| Kurosaki & Hatakeyama | Retrospective cohort study | 2 | 2 | 0 | 2 | 1 | 2 | 1 | 0 | 1 | 2 | 0 | 1 | 15 |
| Barbier et al | Retrospective cohort study | 2 | 2 | 1 | 2 | 1 | 2 | 2 | 0 | 0 | 1 | 0 | 2 | 15 |
| Al Saeedi | Retrospective cohort study | 2 | 2 | 1 | 2 | 1 | 2 | 2 | 0 | 0 | 0 | 0 | 2 | 14 |
