# Supplementary material for: Gastric venous congestion after pancreatic surgery: A systematic review, metanalysis and suggested protocol for assessment and management
Source: Langenbecks Arch Surg. 2026 Apr 25;411(1):159. doi: 10.1007/s00423-026-04049-8 (PMC13249684; doi:10.1007/s00423-026-04049-8)
Supplement: Supplementary file 4 — Supplementary Material 4 (DOCX 16.2 KB) [file 423_2026_4049_MOESM4_ESM.docx]

**Appendix 3: Study quality assessment for case reports and Case series**

| **Author** | **Year** | **Study type** | **1. Does the patient(s) represent(s) the whole experience of the investigator (centre) or is the selection method unclear to the extent that other patients with similar presentation may not have been reported?** | **2. Was the exposure adequately ascertained?** | **3. Was the outcome adequately ascertained?** | **. Were other alternative causes that may explain the observation ruled out?** | **5. Was there a challenge/rechallenge phenomenon?** | **6. Was there a dose–response effect?** | **7. Was follow-up long enough for outcomes to occur?** | **8. Is the case(s) described with sufficient details to allow other investigators to replicate the research or to allow practitioners make inferences related to their own practice?** |
| --- | --- | --- | --- | --- | --- | --- | --- | --- | --- | --- |
| Yamanaka | 2024 | Case Report | Yes | Yes | Yes | No. | No. | No. | Yes | Yes. |
| Sandroussi | 2010 | Case Report | yes | yes | yes | yes | n/a | N/a | no | Yes |
| Reddy et al | 2024 | Case series | Yes | Yes | Yes | yes | yes | N/A | Yes | Yes. |
| Nakamura et al | 2023 | Case series | yes | yes | Yes | partially | NO | N/A | Yes | YES. |
| Strobel et al | 2018 | Technical note | yes | yes | yes | No. | no | No. | N/A | yes |
| Kokoroskos | 2023 | Case report | No | yes | yes | No | no | N/A | Yes | Partially |
| Kagota | 2020 | Case report | Yes | Yes | Yes | Yes | Yes | N/A | Yes | Yes |
| Hackert | 2015 | Technical note | No | yes | Partially | Yes | No | N/A | No | Yes |
| Fernández-Placencia | 2024 | Case report | Yes | Yes | Yes | Yes | No | N/A | Yes | Yes |
