# Supplementary material for: Gastric venous congestion after pancreatic surgery: A systematic review, metanalysis and suggested protocol for assessment and management
Source: Langenbecks Arch Surg. 2026 Apr 25;411(1):159. doi: 10.1007/s00423-026-04049-8 (PMC13249684; doi:10.1007/s00423-026-04049-8)
Supplement: Supplementary file 5 — Supplementary Material 5 (DOCX 16.8 KB) [file 423_2026_4049_MOESM5_ESM.docx]

**Appendix 4: GRADE Assessment of Evidence**

| **Author (Year)** | **Study Design** | **GRADE Rating** | **Comments/Justification** |
| --- | --- | --- | --- |
| Loos (2022) | Retrospective cohort | Moderate | Large cohort (n=585), retrospective design, moderate risk of bias (MINORS 14/24), clear statistical associations with GVC risk |
| Stoop (2023) | Retrospective cohort | Moderate | Good-sized cohort (n=268), higher quality methodology (MINORS 16/24), similar findings to other studies |
| Shiihara (2020) | Retrospective cohort | Moderate | Moderate sample size (n=108), focus on varices rather than direct GVC |
| Barbier (2013) | Retrospective cohort | Moderate | Medium-sized cohort (n=56), limited reporting of GVC-specific outcomes |
| Kurosaki (2005) | Retrospective cohort | Moderate | LGV preservation associated with improved outcomes, moderate risk of bias (MINORS 15/24) |
| Nakao (2018) | Retrospective analysis | Low | Small cohort (n=38), methodological limitations (MINORS 10/24) |
| Al-Saeedi (2021) | Retrospective observational | Low | Very small sample (n=10), successful technique reporting |
| Nakamura (2023) | Case series | Low | Small series (n=5), likely selection bias |
| Reddy (2024) | Case series | Low | Small series (n=3), technical focus |
| Sandroussi (2010) | Case report | Very Low | Single case, novel technique |
| Kagota (2020) | Case report | Very Low | Single case, well-documented technique |
| Kokoroskos (2023) | Case report | Very Low | Single case with complications |
| Fernández-Placencia (2024) | Case report | Very Low | Novel LGV-LAV technique |
| Yamanaka (2024) | Case report | Very Low | Single case with anatomical variant |
| Hackert (2015) | Technical note | Moderate | Technique description without patient outcomes |
| Strobel (2018) | Technical note | Moderate | Technique description without patient outcomes |

*GRADE quality of evidence:*

- *High: Further research is very unlikely to change our confidence in the estimate of effect*
- *Moderate: Further research is likely to have an important impact on our confidence in the estimate of effect and may change the estimate*
- *Low: Further research is very likely to have an important impact on our confidence in the estimate of effect and is likely to change the estimate*
- *Very Low: Any estimate of effect is very uncertain*
