# Supplementary material for: Gastric venous congestion after pancreatic surgery: A systematic review, metanalysis and suggested protocol for assessment and management
Source: Langenbecks Arch Surg. 2026 Apr 25;411(1):159. doi: 10.1007/s00423-026-04049-8 (PMC13249684; doi:10.1007/s00423-026-04049-8)
Supplement: Supplementary file 6 — Supplementary Material 6 (DOCX 27.6 KB) [file 423_2026_4049_MOESM6_ESM.docx]

**Appendix 6:** **Patient Demographics and Clinical Characteristics**

| **Author (Year)** | **Study size (n)** | **Age (years)** | **Sex (M:F)** | **Comorbidities** | **Surgical Indication** |
| --- | --- | --- | --- | --- | --- |
| Kurosaki^16^ (2005) | 55 | 63 (median, range 11-84) | 29:26 | Diabetes mellitus (frequency not specified) | Cancer (96.4%): pancreatic (20), duodenal papilla (16), bile duct (15), other (2); CP (3.6%) |
| Sandroussi & McGilvray^16^ (2010) | 1 | 42 | 0:1 | None reported | Pancreatic adenocarcinoma (100%) |
| Barbier^14^ (2013) | 56 | 59 (median, range 27-81) | 27:29 | Not reported | IPMN (75%), NET (11%), PDAC (9%), metastases (3.6%), CP (1.8%) |
| Hackert^11^ (2015) | N/A | NR | NR | NR | Not specifically reported |
| Nakao^11^ (2018) | 38 | 60.9 (mean, range 39-78) | 20:18 | Not reported | PDAC (68.4%), IPMN (18.4%), metastatic RCC (5.3%), NET (5.3%), CP (2.6%) |
| Strobel^22^ (2018) | N/A | NR | NR | NR | PDAC |
| Kagota ^18^(2020) | 1 | 60 | 0:1 | None reported | Remnant PDAC after previous PD |
| Shiihara ^10^(2020) | 108 | 65 (median) | 66:42 | Not specifically reported | Pancreatic head cancer (100%) |
| Al-Saeedi ^12^(2021) | 10 | 64±8 (mean) | 5:5 | ASA II (60%), III (30%), IV (10%) | PDAC (90%), NET (10%) |
| Loos^1^ (2022) | 585 | 65.9 (median, IQR 58.3-73.8) | 333:252 | ASA I (5.3%), II (53.9%), III (39.4%), IV (1.5%) | PDAC (65.3%), CP (6.0%), cystic lesions (18.1%), NET (4.6%), other (6.0%) |
| Kokoroskos ^17^(2023) | 1 | 74 | 0:1 | Moderate HTN, hypercholesterolemia, past LC | Pancreatic head adenocarcinoma (100%) |
| Nakamura^20^ (2023) | 5 | 65 (median, range 60-77) | 4:1 | Not reported | PDAC (80%), IPMN (20%) |
| Stoop^2^ (2023) | 268 | 69 (median, IQR 59-74) | 143:125 | CVD (49.6%), HTN (41.4%), DM (27.6%), IHD (7.8%), PVD (7.1%), CRI (2.2%) | Cancer (73.1%; PDAC 52.2%), benign (26.9%; IPMN 17.5%) |
| Fernández-Placencia^19^ (2024) | 1 | 49 | 0:1 | None reported | PDAC (100%) |
| Reddy^21^ (2024) | 3 | Case 1: 52; Case 2: 66; Case 3: 49 | 2:1 | Diabetes (1/3 patients) | Case 1: IPMN-associated adenocarcinoma; Case 2-3: PDAC |
| Yamanaka^15^ (2024) | 1 | 65 | 0:1 | None reported | IPMN carcinoma (cT2N0M0 Stage IB) |

*NR = Not reported; CP = Chronic pancreatitis; PDAC = Pancreatic ductal adenocarcinoma; IPMN = Intraductal papillary mucinous neoplasm; NET = Neuroendocrine tumor; CV*

*D = Cardiovascular disease; HTN = Hypertension; DM = Diabetes mellitus; IHD = Ischemic heart disease; PVD = Peripheral vascular disease; CRI = Chronic renal insufficiency; LC = Laparoscopic cholecystectomy; RCC = Renal cell carcinoma*
