# Supplementary material for: Gastric venous congestion after pancreatic surgery: A systematic review, metanalysis and suggested protocol for assessment and management
Source: Langenbecks Arch Surg. 2026 Apr 25;411(1):159. doi: 10.1007/s00423-026-04049-8 (PMC13249684; doi:10.1007/s00423-026-04049-8)
Supplement: Supplementary file 7 — Supplementary Material 7 (DOCX 17.5 KB) [file 423_2026_4049_MOESM7_ESM.docx]

**Appendix 7:** **Anatomic Factors and Recommendations for Prevention of GVC**

| **Author (Year)** | **Key Anatomic and Physiologic Factors** | **Prevention Recommendations** |
| --- | --- | --- |
| Kurosaki (2005) | Division of LGV impairs regional venous drainage; Anastomosis between right and left gastroepiploic veins lacking in ~50% of cases; Antroduodenal portion dependent on fine intramural vascular network after LGV division | Preserve LGV if possible. Perform antecolic duodenojejunostomy with stomach set vertically in left abdomen. |
| Sandroussi (2010) | Removal of all draining veins (left gastric vein, short and posterior gastric veins) compromises venous outflow; Paraesophageal veins inadequate for compensation | LGV-IMV bypass for GVC during radical pancreatic surgery; Preserve IMV in continuity with PV as conduit for gastric venous outflow |
| Barbier (2013) | When pancreatic resection combined with antrectomy and splenectomy, stomach vascularized only by left gastric vessels; LGV sometimes difficult to preserve due to anatomical variations or tumor involvement | Pylorus preservation with right gastric vessel preservation; Venous reconstruction or subtotal gastrectomy for persistent GVC |
| Hackert (2015) | LGV drains both anterior and posterior stomach walls; Two anatomic variants: Type 1 (LGV→PV) and Type 2 (LGV→SpV); Total PD with splenectomy makes LGV the main drainage route | Different approaches based on anatomy: 1) For Type 1: PV resection below LGV-PV junction; 2) For Type 2: Reinsert SpV or reconstruct LGV drainage; 3) For TP with splenectomy: LGV reinsertion if necessary |
| Nakao (2018) | Gastric venous drainage primarily through coronary vein, gastroepiploic veins, and short gastric veins; TP with PV/SMV resection compromises these pathways | Distal gastrectomy for TP when venous preservation impossible; Preserve at least one gastric drainage vein; Consider RGEV-ovarian vein anastomosis; Use preoperative CT angiography |
| Strobel (2018) | Resections of portal venous confluence cause gastric/splenic congestion; Reinsertion of SpV/LGV into portal axis problematic due to distance and tension | Create distal spleno-renal shunt (SpV to left renal vein) as straightforward, effective solution |
| Kagota (2020) | Right gastric and gastroepiploic veins removed in first operation; Left gastroepiploic and short/posterior gastric veins removed in second; Stenosis at LGV-SpV junction; Loss of arc of Barkow during splenectomy | Preserve at least one gastric draining vein; Consider LGV-SpV anastomosis when preservation impossible; Detailed preoperative assessment of drainage veins |
| Shiihara (2020) | Sinistral portal hypertension from PV/SPV obstruction; Risk factors: operative time, blood loss, ligation of SPV, LGV and MCV; 60% incidence of varices when all SPV drainage sacrificed vs. 16.7% when preserved | Preserve SPV venous drainage when possible; If SPV ligation unavoidable, preserve alternative drainage (LGV via PV trunk or IMV via SPV); Avoid sacrificing all SPV drainage pathways |
| Al-Saeedi (2021) | Splenic vein ligation during PD with portal confluence resection compromises venous drainage; When collateral routes (LGV, IMV) also sacrificed, significant risk for GVC and left-sided portal hypertension | Splenorenal shunt as safe, effective measure for GVC; Only necessary for patients showing intraoperative congestion; Technically straightforward (5-15 minutes additional time) |
| Loos (2022) | Normal drainage relies on multiple veins; Post-TP disruption affects right gastric, right gastroepiploic, splenic, and short gastric veins; High-risk factors: splenectomy (OR 2.14) and left gastric vein resection (OR 5.49); Venous outflow obstruction leads to wall edema, petechiae, and hemorrhagic necrosis | Avoid left gastric vein resection unless oncologically necessary; Perform selective splenectomy; Consider left gastric vein reimplantation; Evaluate for GVC via visual inspection; Perform partial gastrectomy if severe GVC detected |
| Kokoroskos (2023) | Disruption of gastric venous drainage after sacrificing both left gastric vein and splenic vein; Left gastric vein drained directly to sacrificed splenic vein | Preserve pancreas with splenic vein or resect splenic vein distal to left gastric-splenic vein junction; Preserve at least one major gastric drainage vein |
| Nakamura (2023) | Gastric venous drainage occurs through LGV, posterior and short gastric veins, and splenic vein; When sacrificed during TP with splenectomy, drainage compromised | Preserve at least one vein for gastric drainage; Adjust gastrectomy extent based on venous preservation; Perform subtotal gastrectomy when all veins sacrificed; Use 3D-CT postoperatively |
| Stoop (2023) | Ligation of splenic vein (86.9%) and left left gastric vein (21.6%) disrupts outflow; PVR associated with 2.1× increased GVC risk; Risk factors: left gastric vein ligation (OR 11.86) and PVR (OR 2.10) | Avoid left gastric vein ligation unless necessary; Consider selective splenectomy; Standardize GVC assessment; Consider venous reconstruction techniques |
| Fernández-Placencia (2024) | GVC from tumor infiltration of LGV and splenic vein causing sinistral portal hypertension; Normal drainage patterns insufficient after LGEV and LGV ligation; LGV is primary outflow route after SpV disconnection | Anastomose LGV to LAV for GVC during RAMPS; Recognize and address GVC intraoperatively; |
| Reddy (2024) | TP with splenectomy requires division of major gastric veins; Splenic vein occlusion disrupts porto-splenic drainage; Distal stomach more affected than proximal due to submucosal venous plexus | Detailed preoperative imaging review; Preserve/reconstruct at least one main gastric vein; Perform "bulldog clamp tests"; Use innovative reconstruction techniques; Consider spleen preservation; Employ microvascular expertise; Consider dual venous reconstruction |
| Yamanaka (2024) | GVC due to increased LGA blood flow after SpA transection in patient with RCHA; Anatomical factors: RCHA branching from SMA led to hemodynamic changes | Total gastrectomy effective but anatomical variations should be recognized pre-operatively |

*LGV = Left gastric vein; NG = Nasogastric; IMV = Inferior mesenteric vein; PV = Portal vein; LGV – left gastric vein; SpV = Splenic vein; TP = Total pancreatectomy; PD = Pancreatoduodenectomy; CT = Computed tomography; RGEV = Right gastroepiploic vein; MCV = Middle colic vein; OR = Odds ratio; LGEV = Left gastroepiploic vein; LAV = Left adrenal vein; RAMPS = Radical antegrade modular pancreatosplenectomy; RCHA = Replaced common hepatic artery; SMA = Superior mesenteric artery; LGA = Left gastric artery; SpA = Splenic artery; GVC = Gastric venous congestion*
